# Biotène Versus HydraSmile for Radiation‐Induced Xerostomia: Randomized Double‐Blind Cross‐Over Study

**DOI:** 10.1002/oto2.70038

**Published:** 2025-01-03

**Authors:** Randall J. Harley, Eve Bowers, Jinhong Li, Mikayla Bisignani, Marci L. Nilsen, Jonas T. Johnson

**Affiliations:** ^1^ Department of Otorhinolaryngology–Head and Neck Surgery University of Pennsylvania Philadelphia Pennsylvania USA; ^2^ Department of Otolaryngology–Head and Neck Surgery University of Miami Miller School of Medicine Miami Florida USA; ^3^ Department of Biostatistics University of Pittsburgh, Graduate School of Public Health Pittsburgh Pennsylvania USA; ^4^ Department of Otolaryngology–Head and Neck Surgery University of Pittsburgh School of Medicine Pittsburgh Pennsylvania USA; ^5^ Department of Acute and Tertiary Care University of Pittsburgh School of Medicine Pittsburgh Pennsylvania USA

**Keywords:** artificial saliva, head and neck cancer, radiation, xerostomia

## Abstract

**Objective:**

This study aims to compare the effectiveness of 2 artificial saliva substitutes (Biotène vs HydraSmile) in the symptomatic management of radiation‐induced xerostomia.

**Study Design:**

Randomized double‐blind cross‐over study.

**Setting:**

Single tertiary care academic institution.

**Methods:**

Included adult patients ≥6 months postradiotherapy (50‐70 gy) for squamous cell carcinoma of the oral cavity, oropharynx, or larynx. The primary endpoint was change in overall subjective xerostomia score from baseline, through use of HydraSmile versus Biotène. Scores were derived from a 100‐point visual analog scale, with higher scores indicating better symptomatic control. Analysis of covariance model was used to regress the difference in after‐treatment measurement between HydraSmile and Biotène, with respect to baseline differences.

**Results:**

A total of 91 participants were included (mean age 63.0 years [SD 9.7]; 85.7% male; 97.8% White). Change in overall xerostomia score with respect to baseline was not significantly different between HydraSmile and Biotène (mean difference 1.24, 95% confidence interval [CI] −2.35 to 4.81). Compared to water alone, both HydraSmile (mean difference 7.45, 95% CI 3.61‐11.29) and Biotène (mean difference 7.24, 95% CI 3.06‐11.43) significantly improved overall xerostomia score. Forty (44%) patients reported a preference for Biotène, 46 (50.5%) preferred HydraSmile, and 5 (5.5%) had no preference. Patients who preferred Biotène did not significantly benefit from HydraSmile, whereas those who preferred HydraSmile did not significantly benefit from Biotène.

**Conclusion:**

Biotène and HydraSmile significantly improved oral dryness among patients with radiation‐induced xerostomia. While neither product demonstrated treatment superiority, individual product preference was predictive of greatest treatment benefit.

Xerostomia is a common side effect of radiotherapy in the setting of head and neck cancer, that is characterized by reduced production and flow of saliva. The clinical presentation typically includes mucosal dehydration and atrophy, reduced lubrication of oral mucosa, microbial colonization and proliferation within the oral cavity, and demineralization of teeth.^1^, ^2^ This can have a substantial impact on a patient's quality of life, causing oral discomfort and pain, disruptions in sleep, dysphonia, dysphagia, hypogeusia, and increased risk of dental caries and oral infections.^1^, ^3^ A prospective study including 288 head and neck cancer patients found that xerostomia affected 5% of patients before radiotherapy, 68% of patients 6 months after radiotherapy, and 60% of patients 24 months after radiotherapy.^4^ In a separate study, Al‐Mamgani et al reported that after a median follow‐up of 44 months postradiotherapy, xerostomia persisted in 33% of their cohort of head and neck cancer survivors.^5^

Despite its prevalence, the management of radiation‐induced xerostomia is challenging, and there are currently no definitive treatments available. Patients commonly self‐manage symptoms through frequent use of water. While this has been shown to improve symptoms, the effects are short‐lived and the resultant urinary frequency can be disruptive, especially during sleep.^1^, ^6^, ^7^ Various therapies, such as saliva substitutes, sialagogues, oral hygiene, muscarinic receptor agonists, surgical transfer of submandibular glands, and salivary stem cell stimulation, can also alleviate symptoms.^8^ Saliva substitutes, such as Biotène, are commonly used for symptomatic management because they are readily available over‐the‐counter, have minimal side effects, and do not require additional surgical procedures.^9^ Manufacturers of saliva substitutes further report that these products can mimic the salivary peroxidase system and introduce other salivary components to boost the mouth's innate antimicrobial and immune mechanisms while providing lubrication and moisture to the oral cavity.^10^, ^11^

HydraSmile is an FDA‐compliant over‐the‐counter artificial saliva spray that is intended for patients with xerostomia. While similar artificial saliva sprays, such as Biotène, have previously been shown to effectively reduce symptoms of radiation‐induced xerostomia in multiple randomized studies, there are currently no randomized controlled trials that include HydraSmile.^10^, ^11^ In this study, we aim to evaluate the effectiveness of HydraSmile compared to Biotène in relieving the symptoms of radiation‐induced xerostomia in a randomized, double‐blind cross‐over design.

## Methods

### Patient Population

**Figure 1:**
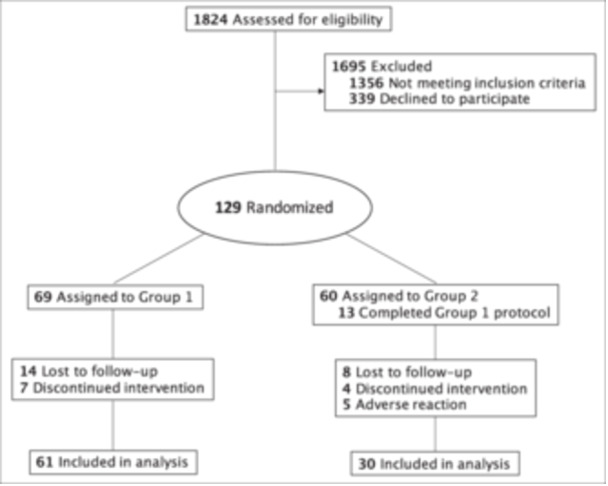
CONSORT flow diagram.

This was a double‐blind, cross‐over study comparing Biotène and HydraSmile among patients with radiation‐induced xerostomia. Patients were recruited from UPMC's Head and Neck Cancer Survivorship Clinic between January 2021 and September 2022. Adult patients with subjective complaints of xerostomia were selected if they were previously diagnosed with squamous cell carcinoma (oral cavity, oropharynx, or larynx) and treated with radiotherapy (between 50 and 70 gray) at least 6 months prior to randomization. Exclusion criteria included any treatment for cancer in the last 6 months (Including surgery, radiation, and chemotherapy), recurrence of cancer, those with other medical conditions associated with xerostomia such as Sjogren's Syndrome, and those using pilocarpine or anticholinergic drugs. A project member explained the study to each participant, who read and signed an informed consent form. See Figure 1 for CONSORT flow diagram. This study was approved by the University of Pittsburgh Institution Review Board.

### Study Design

An authorized third party repackaged the 2 products into opaque bottles labeled “A” or “B.” The contents of each type of spray bottle was not revealed to the research team or the study participants to preserve blinding. At the conclusion of the study, the research team was given the key revealing that Biotène was in bottle A and HydraSmile was in bottle B. Each patient was provided with both oral hydrating sprays (A and B) for use at home. The study was divided into 4 periods: 1 week with only water and no salivary substitute (washout period 1), followed by 2 weeks using one of the provided mouth sprays (mouth spray period 1), followed by 1 week with only water and no salivary substitute (washout period 2), followed by 2 weeks using the other provided mouth spray (mouth spray period 2). Study period length was adapted from previous randomized trials evaluating exogenous xerostomia products.^10^, ^12^ Computer‐generated simple randomization was used to assign patients to 1 of 2 groups:

**Group 1.** Assigned to use product A (Biotène) in mouth spray period 1, followed by product B (HydraSmile) in mouth spray period 2.

**Group 2.** Assigned to use product B (HydraSmile) in mouth spray period 1, followed by product A (Biotène) in mouth spray period 2.

Patients were not permitted to use any other products to treat xerostomia, including chewing gum, hard candy, and lozenges for the entirety of the study. During washout weeks, participants were only permitted to use water for xerostomia relief. During mouth spray periods, participants were instructed to only use the sprays provided and water for xerostomia relief. Participants were permitted to use mouth sprays up to 4 times a day and 4 sprays with each use. Patients were sent a unique link via email to complete an online questionnaire (shown in Supplemental Figure S1, available online) at the end of each of the 4 study periods. The questionnaire included continuous variables derived from the 100 mm visual analog scale (VAS), the gold standard of symptomatic xerostomia evaluation.^10^, ^11^, ^12^, ^13^, ^14^ Higher scores indicate better symptomatic control. At the end of the study, patients were asked “Overall, do you prefer mouth spray A, mouth spray B, or neither mouth spray?”.

### Outcomes

This analysis aimed to compare the relative treatment effect of HydraSmile versus Biotène, as well as evaluate each product's individual benefit compared to water. The primary outcome was change in overall xerostomia score with respect to baseline. The secondary outcomes were change in daytime xerostomia, sleep, speech, swallowing, and taste.

### Statistical Analysis

This study followed a modified intention‐to‐treat design. Patients were required to report which product they used (product A or B) during each mouth spray period in the online questionnaire. During the analysis phase, participants who inadvertently used the products in the wrong order, were reassigned to the appropriate study group based on the protocol they completed. Assuming 1 − β = 0.9 and α = 0.05, a sample size of n = 96 was required to demonstrate a 5‐point change in 100 VAS score. Allowing for a dropout rate of approximately 10%, we aimed to recruit 110 patients.

All statistical analyses were performed using STATA SE 17.0 for Mac OS. Descriptive statistics, including proportions, means, and standard deviations (SD), were used to compare demographic and clinical features between treatment groups. The primary and secondary outcomes were reported xerostomia scores derived from the 100‐mm VAS. Washout period scores were used as the baseline comparison for the mouth spray period that directly followed. Carryover effect was tested by unpaired *t*‐test of the sum of outcomes after both treatments, with sequence as the grouping variable.^15^ Period effect was tested by unpaired *t*‐test of the difference in outcomes between Biotène and HydraSmile after both treatments, with sequence as the grouping variable. To evaluate the treatment effect of Biotène and HydraSmile, we used paired *t*‐test to compare the outcome after treatment compared to the corresponding baseline measurements. To investigate the treatment effect of HydraSmile versus Biotène, we followed the recent recommendation for analysis of 2*2 cross‐over trials with 2 baseline measurements by Metcalfe and Mehrotra and implemented the analysis of covariance (ANCOVA) model to regress the difference in after‐treatment measurement between HydraSmile and Biotène over the difference of baseline between HydraSmile and Biotène.^16^, ^17^ The intercept term would be the treatment effect of HydraSmile compared to Biotène. In the exit survey, patients indicated which mouth spray (Biotène or HydraSmile) they preferred. A planned subgroup analysis was completed within each preference group to determine the effect of each mouth spray and the difference between them. Secondary end points were not adjusted for multiplicity, and therefore should be interpreted as exploratory hypothesis generating data.

## Results

### Study Cohort

A total of 129 patients were enrolled in the study, of which 38 withdrew. Five patients withdrew after experiencing an adverse effect from HydraSmile (oral burning sensation and/or subjective lingual/labial swelling), 11 patients reported no longer having the capacity to participate due to social or medical factors, and 22 patients were lost to follow‐up. No patients experienced anaphylaxis from either product. The remaining 91 participants completed all intervention activities and were included in the final analysis (mean age 63.0 years [SD 9.7]; 85.7% male [n = 78]; 97.8% White [n = 89]). Thirteen patients who were randomized to Group 2 (Product B [HydraSmile], followed by Product A [Biotène]) inadvertently completed the Group 1 protocol (Product A, followed by Product B). These patients were re‐assigned to Group 1 per our modified intention‐to‐treat study design. The final analysis included 61 patients in Group 1 and 30 patients in Group 2 (Figure 1). The majority of patients were previously irradiated for cancer of the oropharynx (72.5% [n = 66]), while a smaller proportion were treated for cancer of the oral cavity (16.5% [n = 15]) or larynx (11% [n = 10]). The mean radiation dose received was 64.9gy (SD 6.2). See Table 1 for summary of demographics and clinical characteristics.

**Table 1:** Demographics and Clinical Characteristics (n = 91)

	Total	Group 1	Group 2	
Variables	n = 91	n = 61	n = 30	*P* value
Age, y, mean (SD)	63.0 (9.7)	63.3 (9.9)	62.4 (9.3)	.66
Sex, n (%)				1
Male	78 (85.7)	52 (85.2)	26 (86.7)	
Female	13 (14.3)	9 (14.8)	4 (13.3)	
Race, n (%)				1
White	89 (97.8)	60 (98.4)	29 (96.7)	
Black	2 (2.2)	1 (1.6)	1 (3.3)	
Site of primary tumor, n (%)				.88
Oral cavity	15 (16.5)	10 (16.4)	5 (16.7)	
Oropharynx	66 (72.5)	45 (73.8)	21 (70)	
Larynx	10 (11.0)	6 (9.8)	4 (13.3)	
Tumor stage, n (%)				.22
Stage I	17 (18.7)	9 (14.8)	8 (26.7)	
Stage II	13 (14.3)	10 (16.4)	3 (10.0)	
Stage III	17 (18.7)	10 (16.4)	7 (23.3)	
Stage IV	41 (45.0)	31 (50.8)	10 (33.3)	
Unknown	3 (3.3)	1 (1.6)	2 (6.7)	
Radiation dose, gy, mean (SD)	65.0 (6.2)	64.6 (6.3)	65.6 (6.0)	.50
Time since last radiation dose, y, mean (SD)	4.8 (4.3)	4.9 (4.3)	4.6 (4.3)	.75

Abbreviations: gy, gray; n, sample size; SD, standard deviation; y, years.

### Primary Outcome

**Figure 2:**
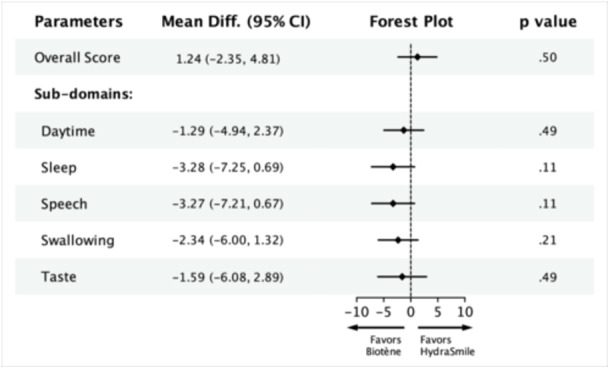
Analysis of covariance model to regress the difference of after‐treatment measurement between HydraSmile and Biotène over the difference in baseline between HydraSmile and Biotène. Level of significance *P* < .05. See Supplemental Figure S1, available online for parameter descriptions. 95% CI, 95% confidence interval; Mean Diff., mean difference.

**Figure 3:**
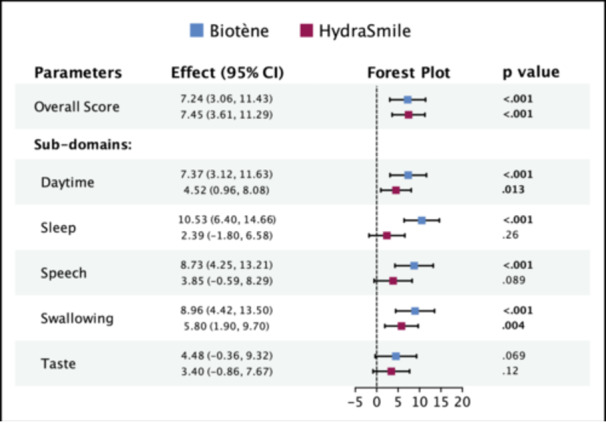
Treatment effect of Biotène and HydraSmile measured as mean difference in visual analogue scale scores between baseline and end of treatment period. Level of significance *P* < .05. See Supplemental Figure S1, available online for parameter descriptions. 95% CI, 95% confidence interval.

Following the protocol outlined in the methods section, we found that there was no significant carryover effect or period effect for any of the 6 parameters evaluated (Supplemental Table S1, available online). At the conclusion of the study, there was no difference in overall treatment effect between HydraSmile and Biotène, with respect to baseline (mean difference 1.24, 95% CI −2.35 to 4.81, Figure 2). Both products, however, were individually effective when compared to use of water alone. Participants achieved clinically significant improvements in overall xerostomia score with use of HydraSmile (mean difference 7.45, 95% CI 3.61‐11.29) and Biotène (mean difference 7.24, 95% CI 3.06‐11.43, Figure 3).

### Secondary Outcomes

Across our 5 secondary outcomes, the treatment effects of HydraSmile and Biotène were not statistically distinguishable (Figure 2). In comparison to use of water alone, participants using HydraSmile achieved statistically significant improvement in VAS score for daytime xerostomia (mean difference 4.52, 95% CI 0.96‐8.08) and clinically significant improvement in VAS score for swallow (mean difference 5.80, 95% CI 1.90‐9.70). With the use of Biotène, participants achieved clinically significant improvement in daytime xerostomia (mean difference 7.37, 95% CI 3.12‐11.63), sleep (mean difference 10.53, 95% CI 6.40‐14.66), speech (mean difference 8.73, 95% CI 4.25‐13.21), and swallow (mean difference 8.96, 95% CI 4.42‐13.50). Neither product allowed for improvement in taste compared to water (Figure 3).

### Product Preference

**Figure 4:**
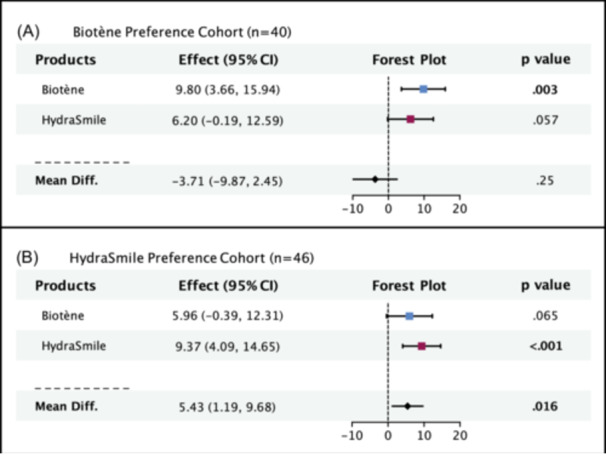
Stratified subgroup analysis showing overall treatment effect of Biotène and HydraSmile within the (A) Biotène preference cohort and the (B) HydraSmile preference cohort. Displayed is the mean difference between baseline and end of treatment period for Biotène and HydraSmile, as well as the difference of after‐treatment measurement between HydraSmile and Biotène over the difference in baseline between HydraSmile and Biotène. Level of significance *P* < .05. See Supplemental Figure S1, available online for parameter descriptions. 95% CI, 95% confidence interval; Mean Diff., mean difference.

In our exit survey, 44% (n = 40) of patients reported a preference for Biotène, 50.5% (n = 46) preferred HydraSmile, and 5.5% (n = 5) had no preference. A subgroup analysis was conducted, in which participants were stratified by product preference. Within the Biotène preference cohort (n = 40), Biotène significantly improved overall xerostomia score (mean difference 9.80, 95% CI 3.66‐15.94, *P* = .003), while HydraSmile did not (mean difference 6.20, 95% CI −0.19‐12.59, *P* = .057; Figure 4A). There was no difference in treatment effect between HydraSmile and Biotène with respect to baseline (mean difference −3.71, 95% CI −9.87‐2.45, *P* = .245; Figure 4A). Within the HydraSmile preference cohort (n = 46), HydraSmile significantly improved overall xerostomia score (mean difference 9.37, 95% CI 4.09‐14.65, *P* < .001), while Biotène did not (mean difference 5.96, 95% CI −0.39‐12.31, *P* = .065; Figure 4B). HydraSmile displayed a greater improvement in overall xerostomia score, with respect to baseline, compared to Biotène (mean difference 5.43, 95% CI 1.19‐9.68, *P* = .016; Figure 4B).

## Discussion

In this study, we found that Biotène and HydraSmile effectively improved symptoms of radiation‐induced xerostomia. While the treatment effects of Biotène and HydraSmile did not significantly differ, our exploratory analysis suggests that Biotène may provide a more comprehensive coverage of the subdomains evaluated. Ultimately, patient preference appeared to be the most important factor in predicting the effectiveness of a given product. Patients who preferred Biotène did not significantly benefit from HydraSmile, whereas those who preferred HydraSmile did not significantly benefit from Biotène. These data emphasize that patients with radiation‐induced xerostomia should be provided with multiple artificial saliva options to determine which works best for them.

### Treatment Effect

Multiple studies have explored the ways in which radiation‐induced xerostomia can reduce patient quality of life.^3^, ^4^ In addition to the lingering oral discomfort, these findings can be understood by considering how xerostomia broadly interferes with activities of daily life such as speech, taste, swallowing, and sleep. Several randomized trials have demonstrated that Biotène, as well as other artificial saliva substitutes, can effectively improve symptoms of xerostomia overall.^7^, ^13^, ^14^ A few of these studies further delineated which components of xerostomia were improved with use of Biotène. Shahdad et al found that, in addition to overall xerostomia relief, Biotène improved swallowing and taste. The authors did not identify a significant improvement in chewing or speech with the use of Biotène.^10^ In a similar study, Warde et al found that Biotène helped improve all domains evaluated, which included oral dryness, oral discomfort, sleep, speech, and swallowing.^11^ In the current study, we found that in addition to improving overall oral dryness, Biotène significantly improved daytime xerostomia, sleep, speech, and swallowing. HydraSmile significantly improved overall oral dryness, daytime xerostomia, and swallowing. HydraSmile was found to provide a positive but nonxsignificant treatment effect for the remaining subdomains evaluated. Perhaps with a larger sample size, HydraSmile would provide a significant benefit with regard to sleep, speech, and taste. While not statistically significant, Biotène tended to outperform HydraSmile within the subdomains tested. In contrast, HydraSmile showed a nonsignificant trend towards outperforming Biotène with regard to overall symptomatic relief. Biotène may be more effective for patients who primarily suffer from disturbances in sleep and speech due to xerostomia, however, the current study does not demonstrate superiority of one product over the other.

### Product Preference

We found that 44% of patients preferred Biotène, 50.5% of patients preferred HydraSmile, and 5.5% of patients had no preference. Interestingly, patients who preferred Biotène did not significantly benefit from HydraSmile, whereas those who preferred HydraSmile did not significantly benefit from Biotène. Additionally, within the HydraSmile preference group, HydraSmile displayed a significantly greater treatment effect compared to Biotène. Currently, there are no other studies that evaluate how xerostomia product preference relates to effectiveness in the setting of xerostomia. While not quantified, many patients reported a preference based on product taste. This could have modified treatment effect by influencing a participant's willingness to use a given xerostomia spray. There are likely additional unmeasured interactions between product ingredients and patient clinical features that have also contributed to this finding. Overall, these results highlight that there is no easy way to predict whether Biotène or HydraSmile will work best for a given patient. If possible, patients should try multiple products to determine which is most effective for them.

### Duration of Effect

While lubricants and saliva substitutes have been shown to reduce symptoms of xerostomia, it has also been reported that these effects are generally short‐lived.^10^, ^11^, ^12^, ^18^ In a study published in 2021, Lung et al measured the mean duration of effect of Biotène spray to be 27 ± 25 min.^19^ Gil‐Montoya et al in a systematic review previously noted that these types of products may not last long enough to improve quality of life meaningfully.^20^ Therefore, rather than testing for the immediate effect of Biotène and HydraSmile after each use, we tailored our study to evaluate how routine use of each product influenced the overall symptomatic burden of xerostomia. Consequently, this study design likely underestimated the immediate treatment effect with each use. We found that both Biotène and HydraSmile provide longitudinal xerostomia relief in addition to immediate relief. We predict that use of these products can improve overall quality of life, however, this hypothesis will require testing in a future study.

### Adverse Effects

Five participants experienced mild adverse effects with use of HydraSmile. While none of the participants experienced anaphylaxis, these adverse reactions may have been allergy related. HydraSmile differs from Biotène in large part due to inclusion of several natural oils (avocado, peppermint, tea leaf, grapefruit, eucalyptus, wintergreen). This stands as another potential benefit of Biotène over HydraSmile. We recommend that patients avoid using HydraSmile if they have known allergies to any of these ingredients and discontinue use if they develop any sort of adverse reaction.

### Limitations

This study is not without limitations. There may be a degree of attrition bias, given that 38 patients were unable to complete the study. Our sample size of 91 fell short of the 96 patients required to have 90% power to detect a 5‐point change in response. Overall, this increases our chance of type II error. Our modified intention‐to‐treat design, in which 13 patients from group 2 were reassigned after completing the group 1 protocol, may have also added bias to our analysis. Many participants found the product labeling (“A” and “B”) confusing and assumed that “A” was intended to be used during the first mouth spray period, and “B” was intended to be used during the second mouth spray period. Given that we found no evidence of a sequence or period effect in this cross‐over study, we feel any bias from this reassignment is minimal. While this study is much larger than similar studies (Warde et al, n = 28; Lopez‐Jornet et al, n = 30), our sample size was not sufficient to adjust for confounding variables in the final analysis. Additionally, we were unable to report objective measurements of salivary function to support our subjective survey data. Finally, the treatment effect of Biotène and HydraSmile was calculated in reference to use of water, which is known to improve oral dryness.^13^, ^14^, ^19^ Therefore, the magnitude of the treatment effect may be underestimated, however, we would expect both products to be affected equally.

## Conclusions

In conclusion, we found that Biotène and HydraSmile effectively improved oral dryness among patients with radiation‐induced xerostomia. Direct comparison of the 2 products revealed a non‐significant difference in treatment effect across all domains evaluated. Therefore, this study did not find one product to be superior to the other. Through subgroup analysis we found that patients who preferred Biotène did not significantly benefit from HydraSmile, whereas those who preferred HydraSmile did not significantly benefit from Biotène. While Biotène and HydraSmile both have the potential to improve oral dryness, we recommend that patients try multiple products to determine which works best for them.

## Author Contributions

**Randall J. Harley**, conceptualization, data curation, investigation, formal analysis, writing—original draft preparation, writing—review and editing; **Eve Bowers**, conceptualization, data curation, investigation, writing—review and editing; **Jinhong Li**, data curation, formal analysis, writing—review and editing; **Mikayla Bisignani**, data curation, writing—review and editing; **Marci L. Nilsen**, conceptualization, data curation, investigation, writing—review and editing; **Jonas T. Johnson**, conceptualization, data curation, funding acquisition, writing—review and editing.

## Disclosures

### Competing interests

TJA Health, LLC, the producer of HydraSmile, provided both products free of charge and covered participant payments. They were not involved in the study design, data collection, data analysis, data interpretation, data reporting, or manuscript production.

### Sponsorships

None.

### Funding source

TJA Health, LLC, the producer of HydraSmile, provided both products free of charge and covered participant payments.

## Supporting information

Supporting information.

Figure S1. Online questionnaire with 100 mm visual analog scale.
