# Supplementary material for: Biotène Versus HydraSmile for Radiation‐Induced Xerostomia: Randomized Double‐Blind Cross‐Over Study
Source: OTO Open. 2025 Jan 3;9(1):e70038. doi: 10.1002/oto2.70038 (PMC11696889; doi:10.1002/oto2.70038)
Supplement: Supplementary file 1 — Supporting information. [file OTO2-9-e70038-s001.docx]

| **Table S1.** Carryover effect and period effect | | |
| --- | --- | --- |
|  | **Carryover Effect** | **Period Effect** |
| Parameters*^a^* | p value | p value |
| Overall | .867 | .084 |
| Daytime | .666 | .557 |
| Sleep | .598 | .324 |
| Speech | .205 | .093 |
| Swallow | .881 | .149 |
| Taste | .843 | .499 |
| *^a^* See Table 1 for parameter description. | | |
