# Supplementary material for: Biotène Versus HydraSmile for Radiation‐Induced Xerostomia: Randomized Double‐Blind Cross‐Over Study
Source: OTO Open. 2025 Jan 3;9(1):e70038. doi: 10.1002/oto2.70038 (PMC11696889; doi:10.1002/oto2.70038)
Supplement: Supplementary file 2 — Figure S1. Online questionnaire with 100 mm visual analog scale. [file OTO2-9-e70038-s002.pdf]

Which study period did you just complete?

- ○ Water Washout 1 (1 week)
- ○ Mouth Spray A (2 weeks)
- ○ Water Washout 2 (1 week)
- ○ Mouth Spray B (2 weeks)

During the last three days, overall, your mouth or tongue was:

Very Dry — Not Dry At all
0 10 20 30 40 50 60 70 80 90 100

In general, during the daytime hours of the last three days, the feeling of your mouth and tongue was:

Extremely Uncomfortable — Comfortable
0 10 20 30 40 50 60 70 80 90 100

During the last three days, due to the dryness of your mouth and tongue, how difficult was it to sleep? Consider such factors as how difficult it was for you to go to sleep, the duration and quality of your sleep, and how often you woke up to drink or urinate.

Very Difficult — Easy
0 10 20 30 40 50 60 70 80 90 100

During the last three days, overall, due to the dryness of your mouth and tongue, how difficult was it to speak without drinking liquids?

Very Difficult — Easy
0 10 20 30 40 50 60 70 80 90 100

During the last three days, overall, due to the dryness of your mouth and tongue, how difficult was it to chew and swallow food?

Very Difficult — Easy
0 10 20 30 40 50 60 70 80 90 100

During the last three days, has your sense of taste been impaired or affected?

Greatly Impaired/Affected — Not Impaired/Affected
0 10 20 30 40 50 60 70 80 90 100
